# Activation of EGFR-DNA-PKcs pathway by IGFBP2 protects esophageal adenocarcinoma cells from acidic bile salts-induced DNA damage

**DOI:** 10.1186/s13046-018-1021-y

**Published:** 2019-01-09

**Authors:** Zhangjian Zhou, Heng Lu, Shoumin Zhu, Ahmed Gomaa, Zheng Chen, Jin Yan, Kay Washington, Wael El-Rifai, Chengxue Dang, Dunfa Peng

**Affiliations:** 1 Department of Surgical Oncology, the First Affiliated Hospital of Xi’an Jiaotong University, 277 Yanta W. Road, Xi’an, 710061 Shaanxi China; 2 Department of Surgery, University of Miami Miller School of Medicine, Miami, FL 33136-1015 USA; 3 Sylvester Comprehensive Cancer Center, University of Miami Miller School of Medicine, Miami, FL 33136-1015 USA; 4 Department of Veterans Affairs, Miami Healthcare System, Miami, FL USA; 5 Department of Gastroenterology, the First Affiliated Hospital of Nanjing Medical University, Nanjing, China; 6 Department of Pathology, Microbiology and Immunology, Vanderbilt University Medical Center, Nashville, TN USA

**Keywords:** IGFBP2, EGFR, DNA-PKcs, DNA damage, Acidic bile salts, Esophageal adenocarcinoma

## Abstract

**Background:**

The incidence of esophageal adenocarcinoma (EAC) is rising rapidly in the US and Western countries. The development of Barrett’s esophagus (BE) and its progression to EAC have been linked to chronic gastroesophageal reflux disease (GERD). Exposure of BE and EAC cells to acidic bile salts (ABS) in GERD conditions induces high levels of oxidative stress and DNA damage. In this study, we investigated the role of insulin-like growth factor binding protein 2 (IGFBP2) in regulating ABS-induced DNA double-strand breaks.

**Methods:**

Real-time RT-PCR, western blot, immunohistochemistry, immunofluorescence, co-immunoprecipitation, flow cytometry, and cycloheximide (CHX) chase assays were used in this study. To mimic GERD conditions, a cocktail of acidic bile salts (pH 4) was used in 2D and 3D organotypic culture models. Overexpression and knockdown of IGFBP2 in EAC cells were established to examine the functional and mechanistic roles of IGFBP2 in ABS-induced DNA damage.

**Results:**

Our results demonstrated high levels of IGFBP2 mRNA and protein in EAC cell lines as compared to precancerous Barrett’s cell lines, and IGFBP2 is frequently overexpressed in EACs (31/57). Treatment of EAC cells with ABS, to mimic GERD conditions, induced high levels of IGFBP2 expression. Knocking down endogenous IGFBP2 in FLO1 cells (with constitutive high levels of IGFBP2) led to a significant increase in DNA double-strand breaks and apoptosis, following transient exposure to ABS. On the other hand, overexpression of exogenous IGFBP2 in OE33 cells (with low endogenous levels of IGFBP2) had a protective effect against ABS-induced double-strand breaks and apoptosis. We found that IGFBP2 is required for ABS-induced nuclear accumulation and phosphorylation of EGFR and DNA-PKcs, which are necessary for DNA damage repair activity. Using co-immunoprecipitation assay, we detected co-localization of IGFBP2 with EGFR and DNA-PKcs, following acidic bile salts treatment. We further demonstrated, using cycloheximide chase assay, that IGFBP2 promotes EGFR protein stability in response to ABS exposure.

**Conclusions:**

IGFBP2 protects EAC cells against ABS-induced DNA damage and apoptosis through stabilization and activation of EGFR - DNA-PKcs signaling axis.

**Electronic supplementary material:**

The online version of this article (10.1186/s13046-018-1021-y) contains supplementary material, which is available to authorized users.

## Background

Over the past few decades, the incidence of esophageal adenocarcinoma (EAC) has increased rapidly in the United States and Western countries [1, 2]. Abnormal exposure of esophageal cells to a mixture of acid and bile salts in patients with chronic gastroesophageal reflux disease (GERD) is a major risk factor for the development of pre-malignant Barrett’s esophagus (BE) and its progression to EAC [3, 4]. Previous studies have shown that exposure to acidic bile salts (ABS) induces DNA damage in BE and EAC cells [5–7]. Accumulation of unrepaired DNA damage in cells can lead to massive genomic instability that can mediate cell death [8]. To maintain DNA damage at tolerable sublethal levels, cancer cells must acquire adaptive pro-survival protective mechanisms.

DNA-dependent protein kinase, catalytic subunit (DNA-PKcs) is an enzyme encoded by PRKDC in humans [9]. It contributes to the repair of DNA double-strand breaks (DSBs) by accessing broken ends of DNA in combination with the other two DNA-binding factors, Ku70 and Ku80 [10]. This complex serves as a molecular scaffold for recruiting DNA repair factors to DNA strand breaks, such as XRCC4 and DNA ligase IV [11]. The kinase activity of DNA-PKcs is required for the non-homologous end joining (NHEJ) pathway of DNA repair, which rejoin double-strand breaks [12–14]. Phosphorylation at Thr2609 of DNA-PKcs plays a key role in NHEJ [15, 16]. Earlier reports have shown that epidermal growth factor receptor (EGFR) plays an important role in the regulation of DNA-PKcs activity in response to radiation or anti-cancer drugs that induce DNA damage [17, 18]. In addition, EGFR nuclear localization is required for modulation of the repair of cisplatin and ionizing radiation-induced DNA damage [17–19].

Insulin-like growth factor binding protein 2 (IGFBP2) is a member of the IGFBP’s family which shares cysteine-rich amino- and carboxyterminal domains for the IGF-binding site [20]. High levels of IGFBP2 have been detected in patients’ sera of some cancers with poor prognostic outcome [21, 22]. In addition to its functions as a secretory protein, IGFBP2 intracellular oncogenic functions promote cancer cell proliferation, invasion, metastasis and drug resistance [23–25]. Interestingly, IGFBP2 protein has a nuclear localization signal sequence, which is required for its nuclear translocation to regulate cell functions or signaling pathways [26, 27]. IGFBP2 overexpression in EAC is associated with drug resistance [28]. However, its signaling and functional role in response to bile salts-induced DNA damage in esophageal tumorigenesis have not been studied.

In the present study, we investigated the expression, signaling and functional roles of IGFPB2 in response to bile salts-induced DNA damage in esophageal tumorigenesis. We detected nuclear localization and associations of IGFPB2 with EGFR-DNA-PKcs in response to acidic bile salts, a mechanism that promotes DNA damage repair and esophageal cell survival in response to the toxic effects of acidic bile salts.

## Methods and materials

### Cell culture

Three immortalized cell lines originated from Barrett’s esophagus, BAR10T (kindly provided by Dr. Rhonda Souza), CPA and CPB (ATCC, Manassas, VA, USA), were cultured with DMEM/F12 (GIBCO, New York, NY, USA), supplemented with 5% fetal bovine serum (FBS, GIBCO), 1% penicillin/streptomycin (GIBCO), 0.4 μg/ml hydrocortisone (Sigma-Aldrich, Saint Louis, MO, USA), 20 mg/L adenine hydrochloride hydrate (Sigma-Aldrich), 140 μg/ml bovine pituitary extract (Thermo Fisher Scientific, Waltham, MA, USA), Insulin-transferrin-sodium selenite media supplement (Sigma-Aldrich) and 20 ng/ml recombinant epidermal growth factor (Sigma-Aldrich). EAC cell lines, FLO1 (ATCC), OE19 (Sigma-Aldrich), SK-GT4 (kindly provided by Dr. Xiaochun Xu at MD Anderson), ESO26 (Sigma-Aldrich) and OE33 (kindly provided by Dr. David Beer) were cultured in DMEM or RPMI 1640 medium (GIBCO), supplemented with 10% FBS and 1% penicillin/streptomycin. All cell lines were grown at 37 °C in 5% CO_2_. Cell lines were authenticated by Genetica DNA Laboratories using short tandem repeat profiling (Genetica DNA Laboratories, Burlington, NC, USA). Cells used were from the stocks immediately after authentication and cultured less than 6 months. Mycoplasma was tested periodically using the qRT-PCR method (Southernbiotech, Birmingham, AL, USA).

### Chemicals

A bile salts cocktail, consisting of an equal molar mixture of sodium salts of glycocholic acid, taurocholic acid, glycodeoxycholic acid, deoxycholic acid (CALBIOCHEM, La Jolla, CA, USA), and glycochenodeoxycholic acid (Sigma-Aldrich), was prepared to reflect the mixture of bile acids in the distal esophagus during GERD, as previously reported [29, 30]. In all experiments, 200 μM final concentration of the bile salts cocktail (40 μM of each of the above bile acids) in pH 4 medium was used.

### Organotypic 3D culture

Organotypic 3D reconstruction cultures were performed as previously described [31, 32]. Briefly, human esophageal fibroblasts (ScienCell, Carlsbad, CA, USA) were seeded into a 3D matrix (75,000 cells/well) containing collagen I (Corning, Tewksbury, MA, USA) and Matrigel (BD Biosciences, Franklin Lakes, NJ, USA) and were incubated for 7 days at 37 °C. Following incubation, FLO1 and OE33 cells were seeded (500,000 cells/well) on top of esophageal fibroblast matrix. Cultures were incubated for an additional 14 days. Cells were then treated with ABS (pH 4, 200 μM) for 30 min then recovered for 1 h. Cultures were harvested, fixed in 4% paraformaldehyde for 1 h, and then transferred to 70% ethanol until pathological process. Sectioning of paraffin-embedded blocks and H&E staining were processed in the Pathology Research Resource at the University of Miami.

### Plasmids and siRNA transfection

The IGFBP2 expression plasmid was designed and obtained from Vectorbuilder (https://en.vectorbuilder.com/). IGFBP2 siRNAs were purchased from Santa Cruz Biotechnology (sc-37,195; Dallas, TX, USA) and Dharmacon (SMARTpool; Lafayette, CO, USA). The siRNA target sequences are provided in Additional file 1: Table S1. FLO1 and OE33 cells were seeded at 2 × 10^5^ cells/well in 6-well plates 24 h before transfection. 40 nM siRNA or 1.5 μg plasmid/well were transfected using the Lipofectamine 3000 Transfection kit (ThermoFisher).

### Quantitative real time RT-PCR

Total RNA was isolated using the Direct-zol RNA MiniPrep Plus kit (ZYMO Research, Irvine, CA, USA). Single-stranded complementary DNA was synthesized using the iScript cDNA Synthesis kit (Bio-Rad, Hercules, CA, USA). Quantitative real time RT-PCR (qRT-PCR) was performed using the CFX Connect real-time system (Bio-Rad) with the threshold cycle number determined by CFX manager 3.0 software. The primer sequences for IGFBP2 are GCGAGGGCACTTGTGAGA (forward) and CCAACATGTTCATGGTGCTG (reverse). The primer sequences for EGFR are CCAGTATTGATCGGGAGAGC (forward) and TGCCTTGGCAAACTTTCTTT (reverse). Reactions were performed in triplicate. The relative mRNA expression fold was normalized to the average value of the reference gene, HPRT1, of the same sample as previously described [33, 34].

### Western blotting analysis

Whole cell lysates were extracted using RIPA Lysis Buffer with proteinase and phosphatase inhibitors (Santa Cruz Biotechnology). Extraction of cytoplasmic and nuclear proteins was performed using the Cell Fractionation Kit (Cell Signaling, Danvers, MA, USA). Protein concentrations of lysates were measured by the Pierce™ BCA Protein Assay (ThermoFisher Scientific) using a FLUO Star OPTIMA microplate reader (BMG, Cary, NC). Equal amounts of proteins were loaded onto gradient 8–10% SDS-PAGE and transferred to nitrocellulose membranes (Bio-Rad). Western blotting was performed following standard procedure. The information of antibodies is provided in Additional file 1: Table S2. The ChemiDoc™ XRS+ Imaging System (Bio-Rad) was used to capture western blot signals. Bands’ intensity was measured using Image lab software (Bio-Rad). The quantification of the proteins was normalized to the intensity of β-actin of the same sample.

### Immunofluorescence staining

Approximately 1 × 10^4^ cells were seeded in 8-chamber Slides (Corning) 24 h after siRNA or plasmid transfection. Cells were treated with ABS (pH 4, 200 μM) for 20 min and recovered for designated time points. After the treatment, cells were fixed with fresh-made 4% formaldehyde for 45 min. Then immunofluorescence staining was performed as previously described [35]. Antibodies for IGFBP2 (Cell signaling), EGFR (Invitrogen) and Phospho-DNA-PKcs (Thr2609) (Invitrogen) were incubated at 4 °C for overnight. Alexa fluor-568 conjugated anti-rabbit and Alexa fluor-488 conjugated anti-mouse secondary antibody (Invitrogen) were incubated for 1 h at room temperature. Cells were mounted with DAPI solution. Images were captured using the All-in-one Fluorescence Microscope (BZ-X700) (Keyence, Itasca, IL, USA).

### Flow cytometry analysis of Annexin V

To quantitate ABS-induced apoptosis, flow cytometry analysis of Annexin V were performed using a FITC Annexin V apoptosis detection kit (BD Biosciences, San Jose, CA). EAC Cells were transfected with 40 nM IGFBP2 SMARTpool siRNA or control siRNA (Dharmacon, Lafayette, CO) using LipoJet transfection reagent (SignaGen Laboratories, Rockville, MD). 48 h post transfection, cells were treated with ABS for 30 min then recovered in regular medium for 3 h. Cells then were collected for FITC Annexin V and PI staining according to the manufacturer’s instruction and subjected to flow cytometry analysis at Flow Cytometry Shared Resource at Sylvester Comprehensive Cancer Center.

### Co-immunoprecipitation assay

Protein G magnetic beads (Millipore, Billerica, MA, USA) were used to capture the primary antibodies for IGFBP2, EGFR, and DNA-PKcs according to the supplier’s instruction. IgG from rabbit or mouse sources (Santa Cruz Biotechnology) were used as the negative control. The same amount of protein from each group was incubated with antibody-bound beads at 4 °C overnight with continuous mixing. The beads were washed and re-suspended, on the second day, using NuPAGE™ LDS Sample Buffer (ThermoFisher) suitable for electrophoresis. Denature was performed at 90 °C for 10 min. Western blotting analysis was performed following standard protocols.

### Cycloheximide (CHX) chase assay

Cycloheximide (CHX) is a protein synthesis inhibitor in eukaryotes, which is used in cell biology to determine the half-life of a given protein [36, 37]. FLO1 cells with IGFBP2 knockdown and control cells were treated with or without ABS for 20 min and then recovered in full medium with 50 μg/ml CHX. Whole cell lysates were collected and analyzed using anti-EGFR antibody by Western blotting. The bands’ intensities of EGFR and β-actin were measured with Bio-Rad Image lab software. The ratio of EGFR/β-actin was obtained for each sample at different time points.

### Immunohistochemistry assay

Tissue microarrays containing 60 de-identified archival cases of EACs as well as the normal esophagus, and 3 non-dysplastic BE was obtained from Tissue Pathology Core at Vanderbilt University Medical Center, Nashville, TN. Immunohistochemical staining was performed using The IHC Select® Immunoperoxidase Secondary Detection system (MilliporeSigma, Burlington, MA, USA) following manufactory’s instruction. The sections were incubated with the IGFBP2 primary antibody (Cell Signaling) overnight. Immunohistochemical results were evaluated for intensity and frequency of the staining and an index score was applied as previously described [33].

### Statistical analysis

Data are expressed as the mean ± SD for parametric data. One-way ANOVA and Newman-Keuls test were used to compare multiple groups. Unpaired Student *t*-test was performed for two independent variables. All statistical analyses were done using GraphPad Prism 5 software. For all analyses, *P* < 0.05 is considered statistically significant.

## Results

### IGFBP2 is overexpressed and induced by acidic bile salts in EACs

**Fig. 1:**
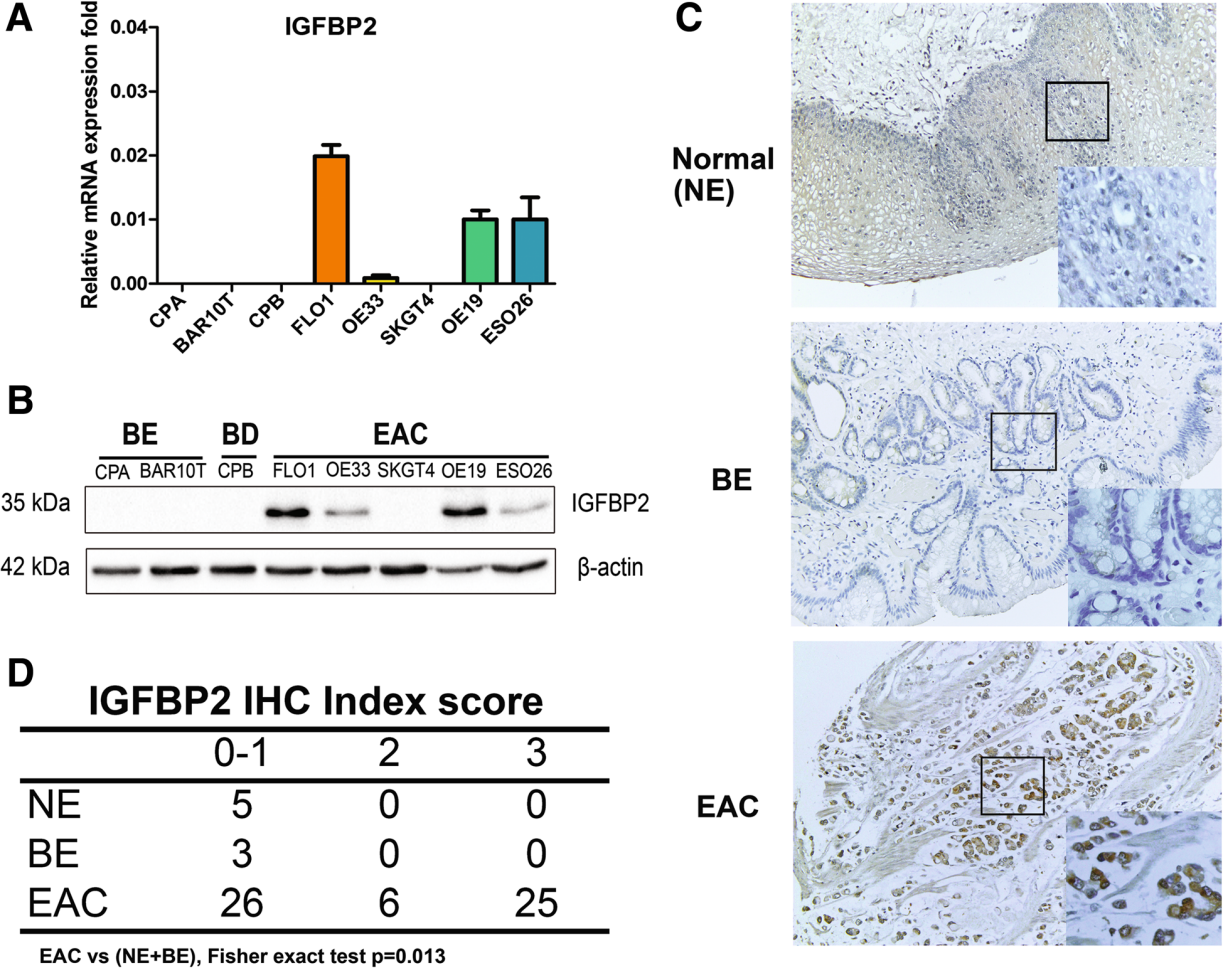
IGFBP2 is upregulated in esophageal adenocarcinomas. **a**, IGFBP2 mRNA level in esophageal cell lines. **b**, IGFBP2 protein expression level in esophageal cell lines. **c**, representative images of IGFBP2 immunohistochemical staining of normal esophagus (NE), Barrett’s esophagus (BE), and esophageal adenocarcinoma (EAC). **d**, Summary of IGFBP2 immunohistochemical staining results in a tissue microarray with 60 EAC samples, 3 EAC samples are missing due to damaged cores. NE, normal esophagus; BE, Barrett’s esophagus; BD, Barrett’s with dysplasia; EAC, esophageal adenocarcinoma; IHC, immunohistochemistry

Using quantitative real-time RT-PCR (qRT-PCR), we detected high levels of mRNA expression of IGFPB2 in several EAC cell lines, whereas its expression is low in precancerous Barrett’s cell lines (CPA, BAR10T) and Barrett’s with dysplasia CPB cells (Fig. 1a). High protein expression levels of IGFBP2 was also present in 4 of 5 EAC cell lines (FLO1, OE33, OE19 and ESO26) (Fig. 1b). Next, we performed immunohistochemical staining of IGFBP2 in a tissue microarray with 60 EAC samples. As shown in Fig. 1c, normal esophageal squamous epithelia (NE) and Barrett’s esophagus (BE) showed negative or weak immunostaining for IGFBP2. However, more than half of EAC tissue samples (31/57) displayed overexpression of IGFBP2 (Fig. 1 c and d, index scores 2 and 3).

**Fig. 2:**
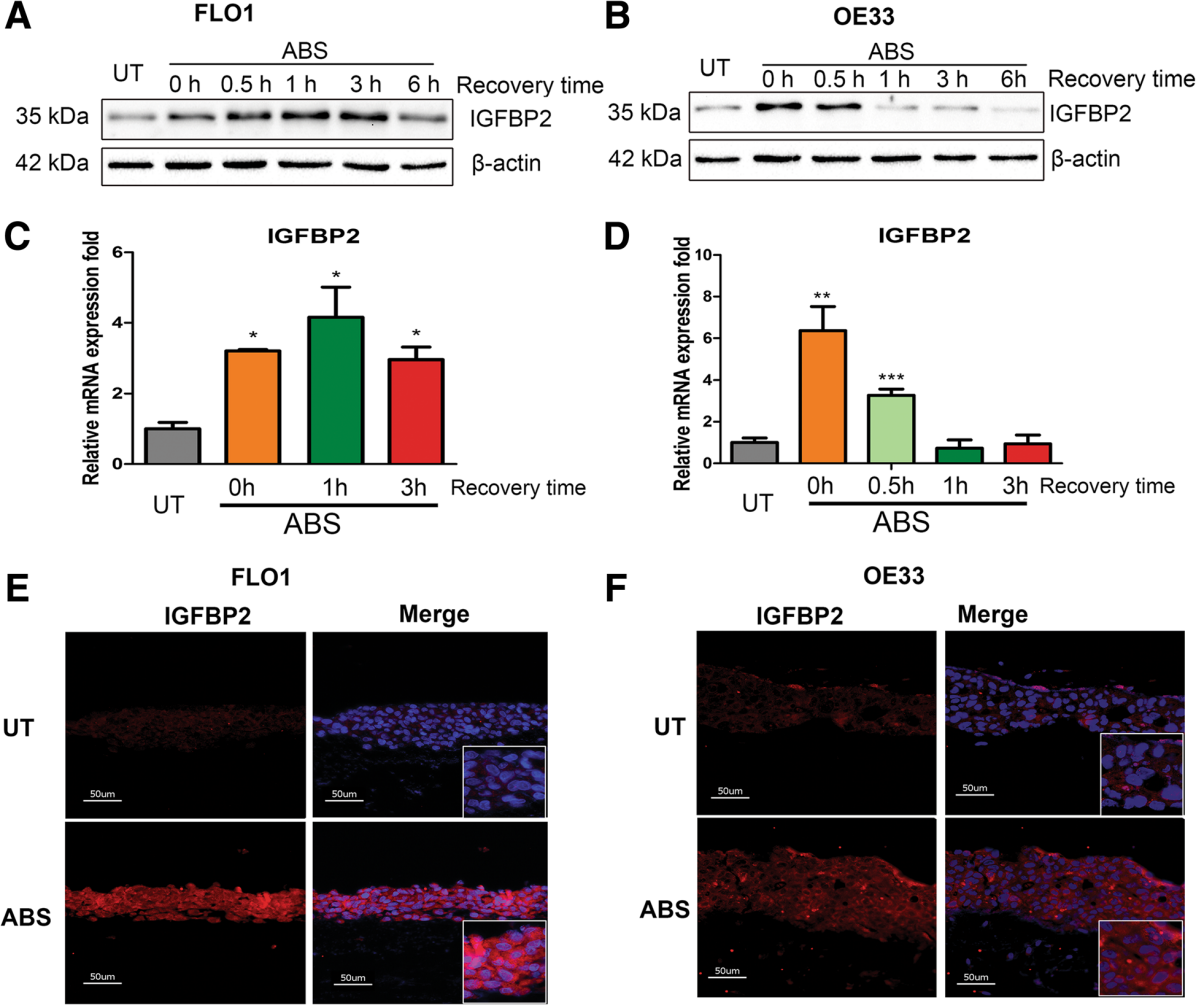
Acidic bile salts induce IGFBP2 in esophageal adenocarcinoma cells. FLO1 and OE33 cells were treated with acidic bile salts (ABS, pH 4, 200 μM) for 20 min and then recovered in regular medium for designated time points. **a** and **b**, Western blotting analysis for IGFBP2 protein expression in FLO1 (**a**) and OE33 (**b**) cells. **c** and **d**, qRT-PCR for IGFBP2 mRNA expression in FLO1 (**c**) and OE33 (**d**) cells. **e** and **f**, immunofluorescence staining of IGFBP2 protein (red) in 3D organotypic cultures of FLO1 (**e**) and OE33 (**f**) cells. DAPI was used to stain nucleus (blue). ABS, acidic bile salts; UT, untreated with ABS; *** *p* < 0.001, ** *p* < 0.01, * *p* < 0.05

Following this finding, we hypothesized that IGFBP2 upregulation is due to adaption to acidic bile salts under GERD conditions. We, therefore, exposed EAC cells to an acidic bile salts cocktail (ABS, pH 4, 200 μM) that closely mimics a reflux episode during GERD [29, 30] . Of note, following transient exposure of 20 min, we detected a remarkable induction of IGFBP2 in protein and mRNA levels in FLO1 (Fig. 2a and c), OE33 (Fig. 2b and d) and OE19 (Additional file 2: Figure S1) cells. Similarly, ABS exposure also induced protein and mRNA expression of IGFBP2 in two Barrett’s cell lines, CPA and BAR10T (Additional file 2: Figure S2). It has been reported that some MiRNAs (such as MiR-126) target IGFBP2 [38, 39] and bile acids induce degradation of some MiRNAs [40]. Therefore, we examined several MiRNAs known regulating IGFBP2 after ABS exposure. Indeed, ABS induced significant downregulation of MiR-126 in both OE33 and FLO1 cells (Additional file 2: Figure S3), suggesting that MiR-126 may play an important role in regulation of IGFBP2 in response to ABS.

To mimic the physiological and pathological conditions of GERD, we cultured EAC cells together with esophageal fibroblasts in a 3D organotypic culture model. We exposed 3D cultured EAC cells to ABS, and examined IGFBP2 protein expression levels using immunofluorescence assay. Data shows that IGFBP2 levels were clearly enhanced by ABS in both FLO1 (Fig. 2e) and OE33 cells (Fig. 2f) cells. The HE staining images of the organotypic cultures for FLO1 and OE33 cells are provided in Additional file 2: Figure S4.

### IGFBP2 protects EAC cells from acidic bile salts-induced DNA double-strand breaks and apoptosis

**Fig. 3:**
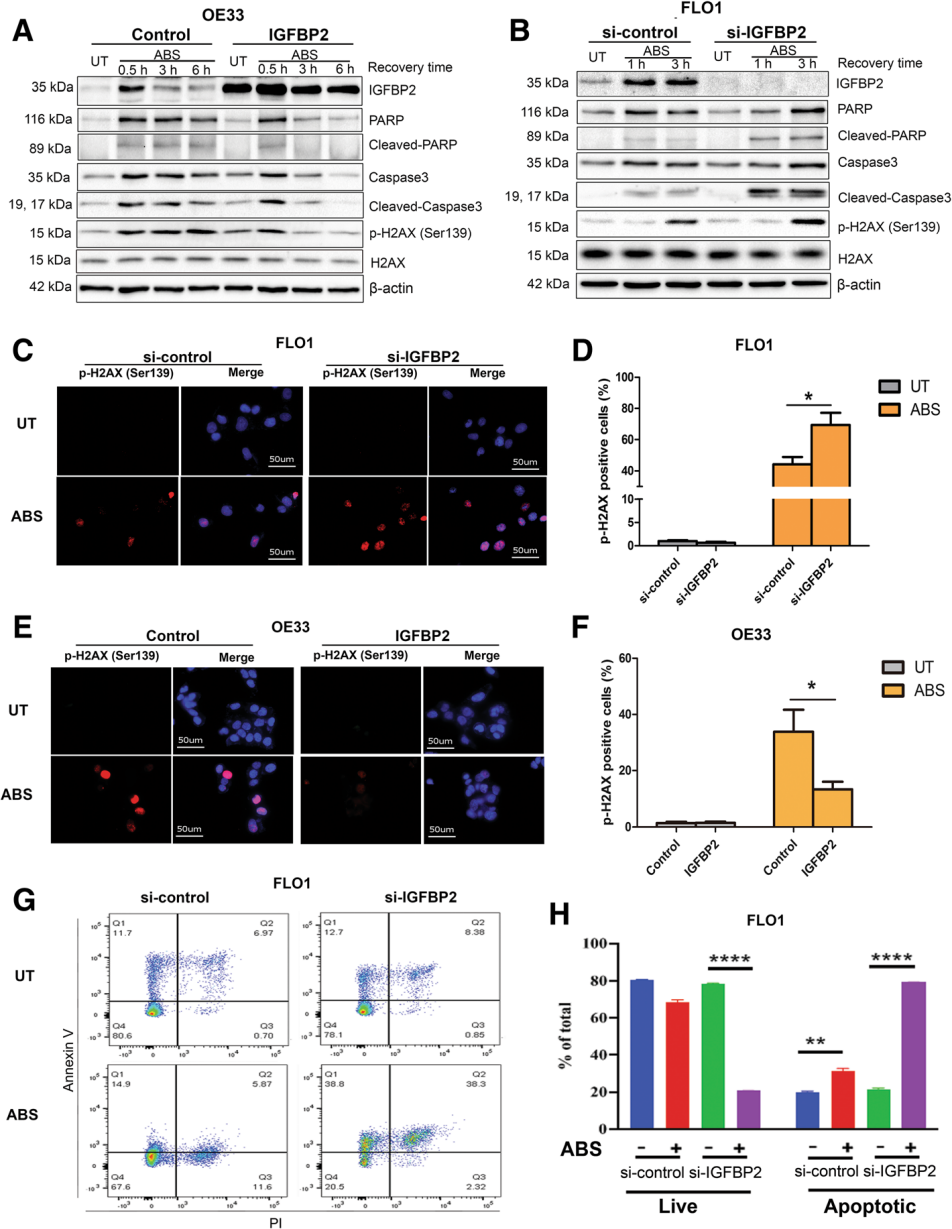
IGFBP2 protects esophageal adenocarcinoma cells from acidic bile salts-induced DNA double strand breaks and apoptosis. **a**, OE33 cells with IGFBP2 overexpression (IGFBP2) and control cells were treated with ABS (pH 4, 200 μM) for 20 min and then recovered for the designated time points. Western blot analysis was used to detect levels of IGFBP2, caspase3, PARP and H2AX. **b**, FLO1 cells with IGFBP2 knockdown (si-IGFBP2) and control cells (si-control) were treated with ABS for 20 min and then recovered for designated time points. Western blot analysis was used to detect levels of IGFBP2, caspase3, PARP and H2AX. **c** and **d**, FLO1 cells with IGFBP2 knockdown and control cells were treated with ABS for 20 min and then recovered for 3 h. Immunofluorescence staining for p-H2AX (Ser139, red) was performed. DAPI (blue) was used to stain cell nucleus. Quantification of positive p-H2AX cells was shown in D**. e** and **f**, OE33 cells with IGFBP2 overexpression and control cells were treated with ABS for 20 min and then recovered for 3 h. Immunofluorescence staining for p-H2AX (Ser139, red) was performed. DAPI (blue) was used to stain cell nucleus. Quantification of positive p-H2AX cells was shown in **f. g** and **h**, flow cytometry analysis of Annexin V positive cells in FLO1 cells with IGFBP2 knock down or control siRNA, treated with ABS for 30 min and recovered for 3 h. **g** shows representative flow cytometry profiles and **h** displays bar graph of live and apoptotic cells. ABS, acidic bile salts; UT, untreated with ABS. * *p* < 0.05, ** *p* < 0.01, **** *p* < 0.0001

Previous studies have indicated that ABS can induce DNA damage as well as apoptosis in esophageal epithelial cells [6, 41]. Therefore, we treated EAC cells with ABS (pH 4, 200 μM) and detected an increase in double strand DNA breaks, as measured by γH2AX (p-H2AX, Ser139), and apoptosis, displayed as cleaved-PARP and cleaved-caspase 3 (Additional file 2: Figure S5, A (FLO1), B (OE33) and Additional file 2: Figure S1 (OE19). Immunofluorescence staining assay confirmed nuclear γH2AX in ABS-treated FLO1 and OE33 cells (Additional file 2: Figure S5, C and D). We next, investigated the role of IGFBP2 upregulation in protecting cancer cells from excessive ABS-induced DNA damage and apoptosis. Overexpression of IGFBP2 in OE33 cells, with relatively low endogenous level, decreased ABS-induced γH2AX, cleaved-PARP and cleaved-caspase 3 (Fig. 3a). To confirm the role of IGFBP2, we knocked down IGFBP2 expression in FLO1 cells, with high endogenous level, and treated cells with ABS. As shown in Fig. 3b, ABS induced higher levels of γH2AX, cleaved-PARP and cleaved-caspase 3 in IGFBP2 knockdown cells, as compared with the control cells. To exclude the possibility of off-target effect of siRNA, we repeated the same experiment using a second IGFBP2 siRNA from a different supplier and obtained similar results (Additional file 2: Figure S6). For further validation, we confirmed these results by using immunofluorescence staining of γH2AX in both FLO1 (Fig. 3c and d) and OE33 (Fig. 3e and f) cells. We also performed flow cytometry analyses of annexin V, confirming that knockdown of IGFBP2 significantly promoted ABS-induced apoptotic cell death in FLO1 cells (Fig. 3g and h), as well as in OE33 cells (Additional file 2: Figure S7).

### IGFBP2 is required for acidic bile salts-induced activation of EGFR-DNA-PKcs pathway in EAC cells

**Fig. 4:**
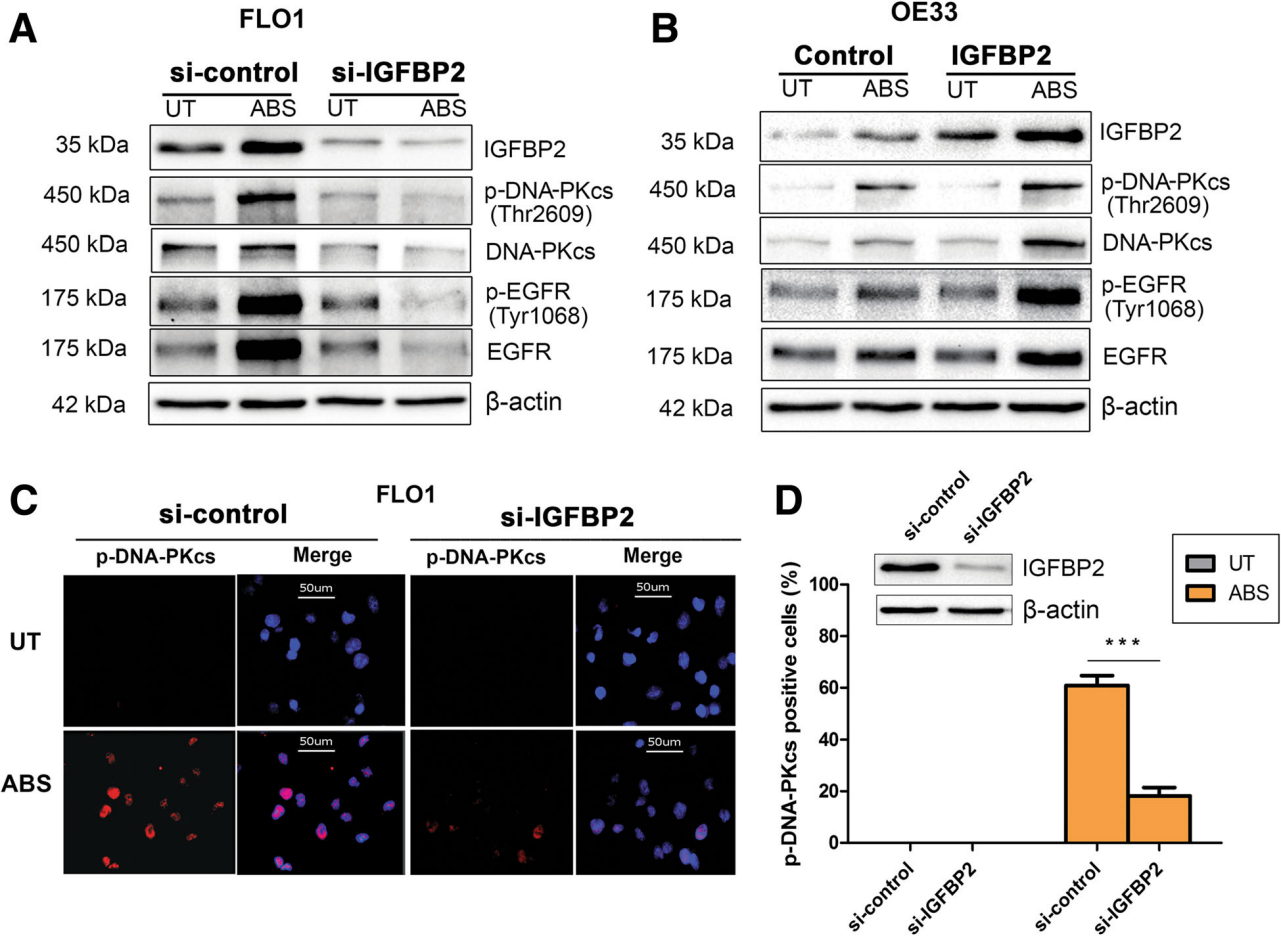
IGFBP2 is required for acidic bile salts-induced EGFR and DNA-PKcs activation in EAC cells. **a**, FLO1 cells with IGFBP2 knockdown (si-IGFBP2) and control cells were treated with acidic bile salts (ABS, pH 4, 200 μM) for 20 min and then recovered for 0.5 h. Whole cell lysates were applied in western blot analysis to detect p-EGFR (Tyr1068) and p-DNA-PKcs (Thr2609). **b**, OE33 cells with IGFBP2 overexpressing and control cells were treated with ABS for 20 min and then recovered for 0.5 h. Whole cell lysates were used for western blot analysis to detect p-EGFR (Tyr1068) and p-DNA-PKcs (Thr2609). **c** and **d**, FLO1 cells with IGFBP2 knockdown and control cells were treated with ABS for 20 min and then recovered for 0.5 h. Immunofluorescence staining for p-DNA-PKcs (Thr2609) was performed. Quantification of positive p-DNA-PKcs cells was performed using image J and presented as a percentage of positive cells (**d**). *** *p* < 0.001

To determine whether EGFR-DNA-PKcs pathway is involved in ABS-induced DNA damage repair, we examined the levels of phosphorylated EGFR (Tyr1068) and phosphorylated DNA-PKcs (Thr2609), which are representatives of enzymatic activity of EGFR and DNA-PKcs, respectively [17, 42]. Our data showed that phosphorylation of EGFR (Tyr1068) and DNA-PKcs (Thr2609) was markedly induced by ABS in FLO1 and OE33 cells (Additional file 2: Figure S8). To determine the role of IGFBP2 in regulating the EGFR-DNA-PKcs DNA damage repair pathways, we knocked down IGFBP2 in FLO1 cells and exposed cells to ABS. As shown in Fig. 4a, knockdown of IGFBP2 attenuated ABS-induced phosphorylation of EGFR and DNA-PKcs, indicating reduced DNA repair activity of DNA-PKcs. To validate these results, we overexpressed IGFBP2 in OE33 cells and treated cells with ABS. As shown in Fig. 4b, IGFBP2 overexpression induced more phosphorylation of DNA-PKcs and EGFR in OE33 cells, following exposure to ABS, as compared to control cells. Immunofluorescence staining assay further confirmed a decrease in nuclear phospho-DNA-PKcs, following ABS exposure in IGFBP2 knockdown FLO1 cells, as compared to control cells (Fig. 4c and d). These data indicate that IGFBP2 is required for EGFR and DNA-PKcs activation in conditions of ABS exposure in EAC cells.

### IGFBP2 is required for nuclear accumulation and activation of EGFR and DNA-PKcs in EAC cells in response to ABS

**Fig. 5:**
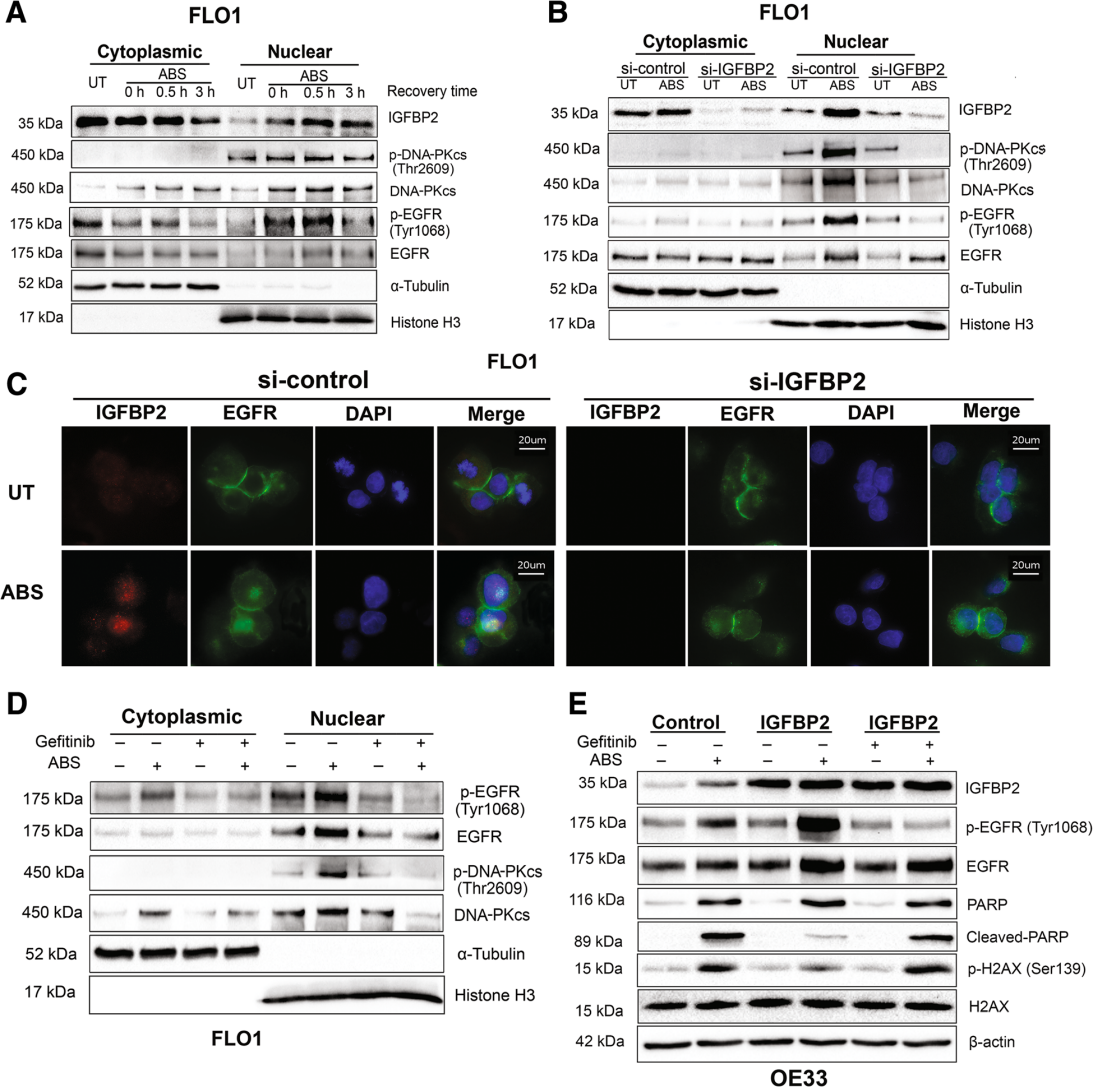
IGFBP2 is required for acidic bile salts-induced EGFR nuclear accumulation to activate DNA-PKcs in EAC cells. **a**, FLO1 cells were treated with acidic bile salts (ABS, pH 4, 200 μM) for 20 min and recovered for 0.5 h and 3 h. Cytoplasmic and nuclear fractions were analyzed using western blot analysis. Data shows that ABS induces IGFBP2 nuclear translocation as well as accumulation of p-EGFR. **b**, FLO1 cells with IGFBP2 knockdown (si-IGFBP2) and control cells were treated with ABS for 20 min and recovered for 0.5 h. Cytoplasmic and nuclear fractions were analyzed by using western blot analysis. Data displays attenuated nuclear accumulation of IGFBP2, p-EGFR, and p-DNA-PKcs, following knockdown of IGFBP2 and ABS treatment. **c**, FLO1 cells with IGFBP2 knockdown and control cells were treated with ABS for 20 min and recovered for 0.5 h. Immunofluorescence staining for IGFBP2 (red) and EGFR (green) was performed. DAPI (blue) was used to stain the nuclei. **d**, FLO1 cells were treated with EGFR tyrosine kinase inhibitor, gefitinib (10 μM) for 24 h. Then cells were exposed to ABS for 20 min and recovered for 0.5 h. Cytoplasmic and nuclear fractions were analyzed by using western blotting. **e**, OE33 cells with IGFBP2 overexpression were treated with and without EGFR tyrosine kinase inhibitor, gefitinib (10 μM) for 24 h. Then cells were treated with ABS for 20 min and recovered for 3 h. Cytoplasmic and nuclear fractions were analyzed by using western blotting

Earlier reports indicate that EGFR translocases into the nucleus, forms a complex with DNA-PKcs, and induces DNA-PKcs phosphorylation, following cisplatin or radiation-induced DNA damage [17, 19]. To examine if IGFBP2 regulates the translocation of EGFR upon ABS treatment, we extracted cytoplasmic and nuclear fractions from ABS-treated FLO1 and OE33 cells. As shown in Fig. 5a, EGFR and DNA-PKcs as well as IGFBP2 translocated into the nucleus, 30 min after ABS treatment in FLO1 cells. In addition, phosphorylated DNA-PKcs and EGFR proteins also accumulated in the nucleus. Similar results were obtained in OE33 cells (Additional file 2: Figure S9). To find whether IGFBP2 mediates EGFR nuclear translocation and, thus, activates DNA-PKcs, we knocked down IGFBP2 in FLO1 cells and treated cells with ABS. As shown in Fig. 5b, IGFBP2 knockdown attenuated ABS-induced nuclear accumulation of total and phospho-EGFR and DNA-PKcs. Immunofluorescence staining assays confirmed nuclear co-localization of IGFBP2 and EGFR in FLO1 cells, following treatment with ABS (Fig. 5c). Nuclear EGFR was hardly visible, following knockdown of IGFBP2.

Earlier studies have suggested that EGFR phosphorylation at Tyr1068 is required for its nuclear translocation in response to cisplatin-induced DNA damage [19, 43]. We, therefore, used tyrosine kinase inhibitor, gefitinib, to examine whether phosphorylation of EGFR is required for its nuclear translocation in response to ABS. As shown in Fig. 5d, treatment with gefitinib attenuated EGFR nuclear accumulation in response to ABS. As expected, less phospho-DNA-PKcs in the nucleus was also observed in gefitinib-treated cells. To confirm that the DNA damage repair function of IGFBP2 is mediated by EGFR, we performed rescue experiments by overexpressing IGFPB2 in OE33 cells, followed by treatment with ABS and gefitinib (Fig. 5e). Overexpression of IGFBP2 enhanced p-EGFR and inhibited ABS-induced cleaved PARP and p-H2AX. However, administration of gefitinib abrogated the protective function of IGFBP2 and impaired ABS-induced phosphorylation of EGFR with a notable increase in the ABS-induced cleaved PARP and p-H2AX levels.

### IGFBP2 forms a complex with EGFR and DNA-PKcs in EAC cells in response to ABS

**Fig. 6:**
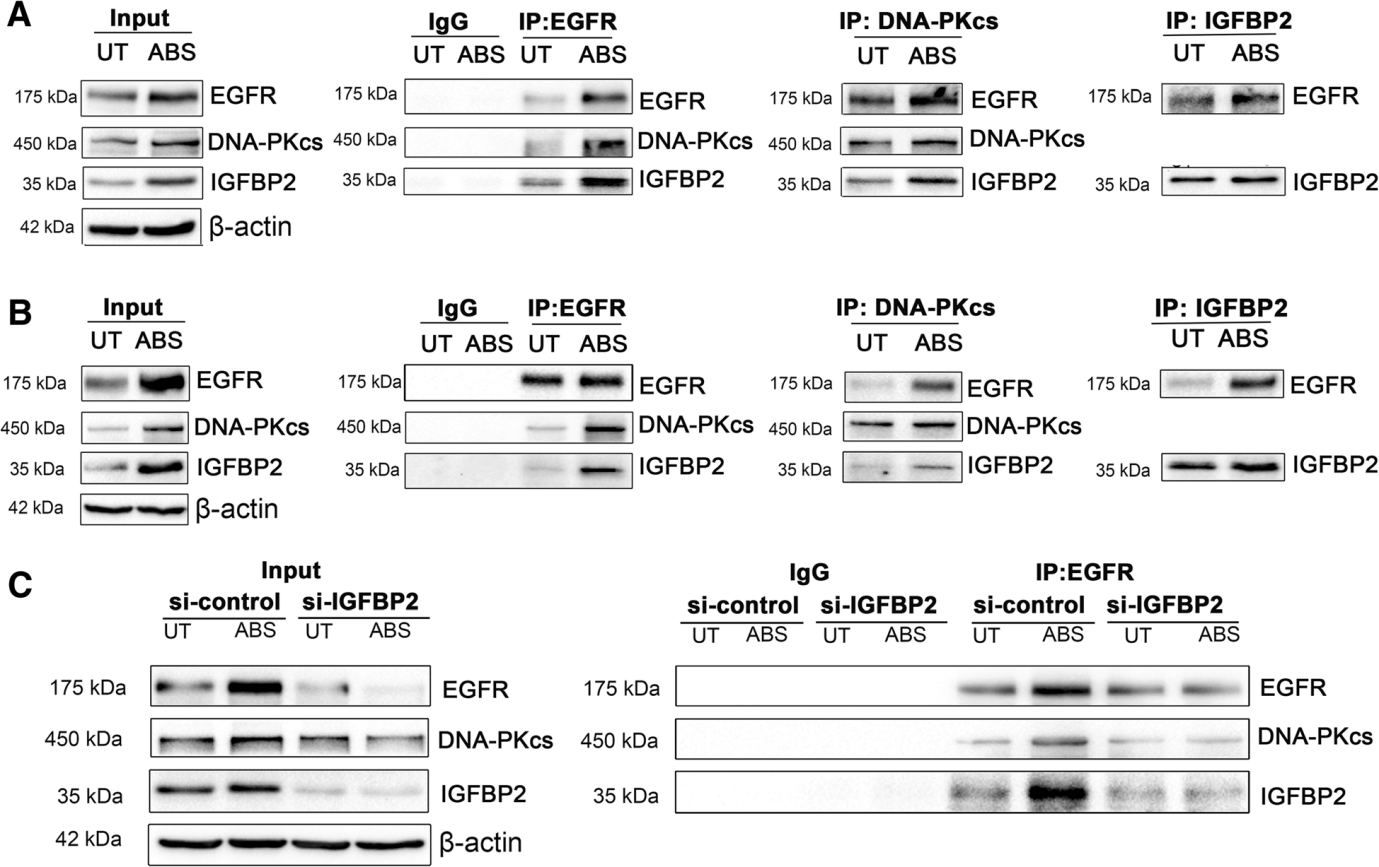
IGFBP2 forms a complex with EGFR and DNA-PKcs in the EAC cells in response to acidic bile salts. **a** (FLO1) and **b** (OE33) cells were treated with acidic bile salts (ABS, pH 4, 200 μM) for 20 min and then recovered for 0.5 h. Co-immunoprecipitation was performed using antibodies against EGFR, DNA-PKcs, and IGFBP2. Westering blot analysis was used to detect the indicated proteins. DNA-PKcs was not detected in IGFBP2 immunoprecipitation blots, possibly due to the large size of DNA-PKcs protein (450 kDa) that cannot be efficiently pulled down by the relatively small IGFBP2 (35 kDa). **c**, FLO1 cells with IGFBP2 knockdown (si-IGFBP2) and control cells were treated with ABS for 20 min and then recovered for 0.5 h. Co-immunoprecipitation was performed using an antibody against EGFR. Westering blot analysis was used to detect the indicated proteins. IgG was used as the negative control. UT, untreated with ABS

Based on our findings, we hypothesized that IGFBP2 regulates EGFR and DNA-PKcs pathway through enhanced binding with this complex, under ABS exposure conditions. We carried out co-immunoprecipitation assays (co-IP) using antibodies against EGFR, DNA-PKcs, and IGFBP2. As shown in Fig. 6a (FLO1) and 6B (OE33), the co-IP assay showed that IGFBP2, EGFR, and DNA-PKcs co-exist in the same protein complex, markedly enhanced after ABS treatment. To prove that IGFBP2 is required for the interaction between EGFR and DNA-PKcs, under ABS exposure conditions, we repeated co-IP for EGFR, following IGFBP2 knockdown and ABS treatment. As shown in Fig. 6c, IGFBP2 knockdown in FLO1 cells led to a remarkable reduction in the interaction between EGFR and DNA-PKcs, as compared with control cells.

### IGFBP2 is required for stabilization of EGFR after ABS treatment in EAC cells

**Fig. 7:**
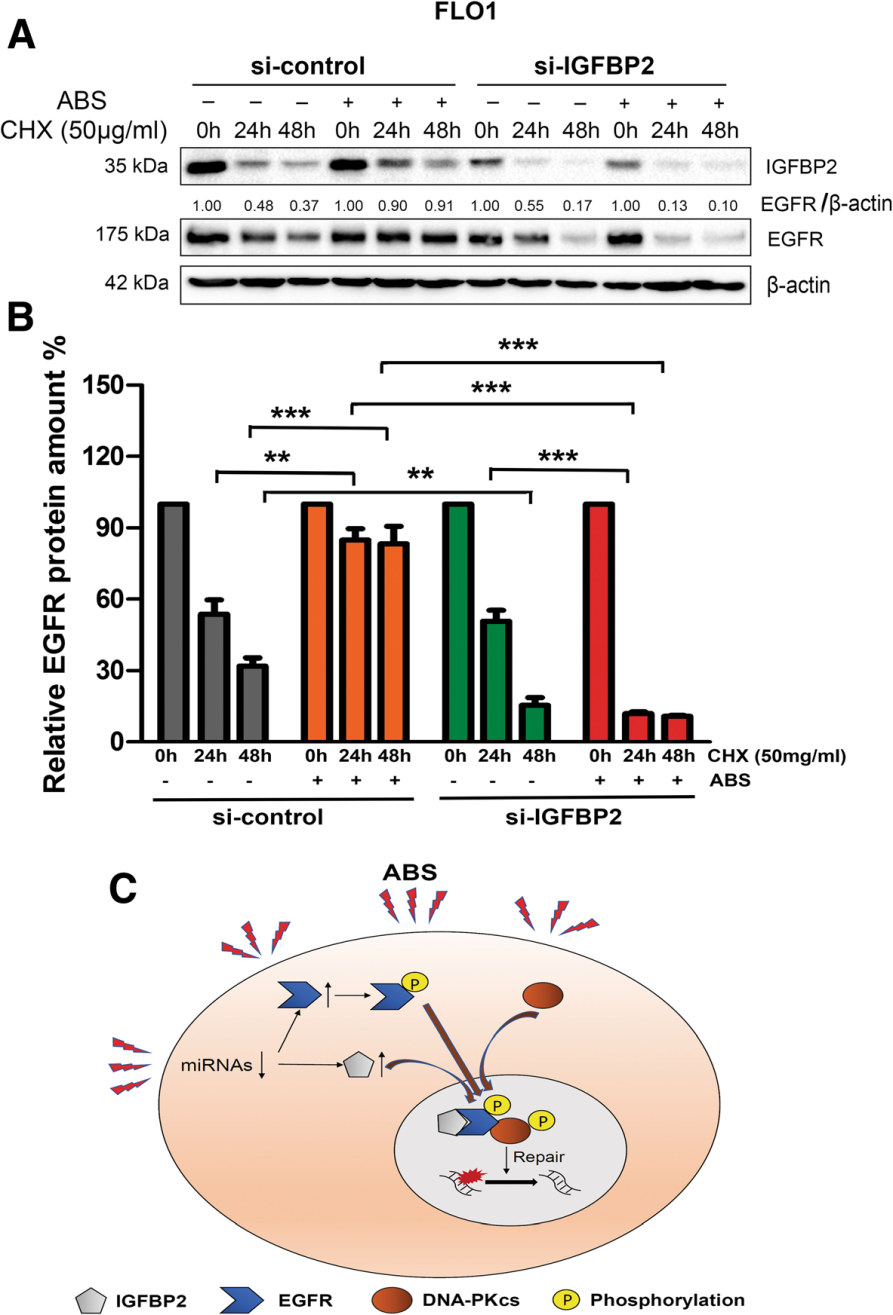
IGFBP2 is required for EGFR stabilization in FLO1 cells after acidic bile salts treatment. **a**, FLO1 cells with IGFBP2 knockdown (si-IGFBP2) and control cells (si-control) were treated with or without acidic bile salts (ABS, pH 4, 200 μM) and then recovered in full medium with cycloheximide (CHX, 50 μg/ml) for the indicated time points. Whole cell lysates were used for western blot analysis to determine the half-life time change of EGFR. **b**, Quantification of relative EGFR protein amounts at different time points (EGFR/β-actin). *** *p* < 0.001, ** *p* < 0.01. **c**, A graphic cartoon summarizing the findings in this study. ABS induces degradation of MiRNAs that targets IGFBP2, leading to upregulation of IGFBP2 and EGFR. IGFBP2 binds to EGFR and stabilizes EGFR protein and promotes EGFR nuclear accumulation. IGFBP2 forms a complex with EGFR and DNA-PKcs. EGFR activates DNA-PKcs to mediate repair of DNA double strand breaks induced by ABS exposure

Because we observed that ABS induced a significant upregulation of EGFR protein, whereas the EGFR induction was impaired in cells with IGFBP2 knockdown (Fig. 4), we examined mRNA levels of EGFR in control and IGFBP2 knockdown FLO1 cells. Knockdown of IGFBP2 did not affect mRNA levels of EGFR without or with ABS treatment (Additional file 2: Figure S10). We therefore hypothesized that the observed differences in EGFR protein levels in IGFBP2 control and knockdown cells are likely due to changes in EGFR protein stability under cellular stress conditions. To confirm this, FLO1 cells with IGFBP2 knockdown and control cells were treated with or without ABS, and then recovered in full medium with cycloheximide (CHX, 50 μg/ml) for 0, 1, 24 and 48 h to block protein synthesis. Western blot assay was used to detect changes in the EGFR protein degradation rate. As shown in Fig. 7, a and b, ABS treatment stabilized EGFR proteins, at least up to 48 h (average EGFR/actin ratio 0.83 in ABS treated cells as compared to 0.32 in ABS untreated si-control cells, *p* < 0.001). Knockdown IGFBP2 alone in FLO1 cells slightly decreased EGFR protein stability as compared to control cells (EGFR/actin ratio 0.32 in si-control cells vs 0.14 in si-IGFBP2 cells at 48 h time point, *p* < 0.01). However, when cells without IGFBP2 (si-IGFBP2) were under stress with ABS treatment, EGFR proteins degraded more rapidly than that without ABS treatment (EGFR/actin ratio 0.12 in cells with ABS vs 0.51 in cells without ABS at 24 h time point, p < 0.001). These data suggest that IGFPB2 plays a role in stabilizing EGFR protein under ABS stress conditions.

## Discussion

Chronic gastroesophageal reflux disease, where acidic bile salts abnormally reflux into the lower esophagus, is a major risk factor for the development of Barrett’s esophagus and EAC [4, 44]. Acidic bile salts induce high levels of oxidative stress that damage cellular components including proteins, lipids, and DNA [45]. This cellular stress environment persists along the stages of BE and its progression to EAC [5, 6, 46]. Therefore, EAC cells must develop adaptation mechanisms to overcome this toxic environment, promote cell survival, and maintain DNA damage below lethal levels. In this study, we discovered that IGFBP2 is expressed at high levels in EAC tissues and cell lines. We also found that IGFPB2 is further induced in both mRNA and protein levels in EAC cells following exposure to ABS. Our findings demonstrate that IGFBP2, under ABS-induced stress condition, promotes stability and nuclear translocation of EGFR and co-exists with EFGR-DNA-PKcs protein complex, essential for DNA repair under stress condition.

IGFBP2 was originally discovered in serum for its binding to IGF1/2, ligands for IGF1R, to maintain activation of the IGF1R signaling pathway [47, 48]. Although IGFBP2 is a secretory protein, IGFBP2 has a nuclear translocation sequence, necessary for its import into the nucleus [26]. Overexpression of intracellular IGFBP2 has been described in several human cancers where it is associated with cellular proliferation, progression and drug-resistance [25–27]. In this study, we detected high levels of IGFPB2 in EAC tissues and in vitro cell models, suggesting a possible role in Barrett’s esophageal tumorigenesis. We focused our studies on the mechanistic relationship between IGFBP2 and ABS, a major etiological risk factor that underlies the biology of EAC. Because acidic bile salts induce high levels of ROS and DNA damage [6, 7], EAC cells must evolve to endure this harsh environment for survival. Our data indicate that IGFBP2 protects against ABS-induced double strand breaks and may thus favor tumor cell survival under reflux conditions.

In response to DNA damage caused by anti-cancer drugs or radiation, DNA-PKcs is phosphorylated at Thr2609, an essential step for its DNA repair capacity [15, 16]. DNA-PKcs recruits other DNA damage repair proteins such as Ku70 and Ku80, XLF, XRCC4 and LigIV to repair damaged DNA [11]. We found that phosphorylation of DNA-PKcs at Thr2609, following ABS, was regulated by levels of IGFBP2. Earlier studies suggested that EGFR binds to DNA-PKcs and induces its phosphorylation [19, 42]. In agreement with this note, our data suggest that IGFBP2 is required for stabilization and nuclear translocation of EGFR, and is required for formation of the EGFR-DNA-PKcs nuclear complex. Of note, we found that the interaction between IGFPB2 and the EGFR-DNA-PKcs complex is markedly enhanced, in response to ABS-induced stress. However, the precise mechanism by which IGFBP2 stabilizes EGFR under ABS stress conditions warrants further investigations.

We postulated that induction of IGFBP2 in response to ABS could be due to regulation of miRNAs. An earlier study has shown that MiR-126 can target IGFBP2 for degradation [38]. Our studies demonstrated that MiR-126 is significantly downregulated in response to ABS, inversely correlating with IGFBP2 levels. This finding provides a plausible mechanism for regulation of IGFBP2 in our system. It is also possible that a similar mechanism may be involved in regulation of EGFR in response to ABS, such as MiR-141 [49]. Of note, downregulation of MiR-141 has been reported in Barrett’s esophagus [50]. However, further studies are needed to validate this mechanism.

IGFBP2 is a secretory protein with high levels in the serum of patients with several types of cancer [21, 22, 51]. High levels of IGFBP2 in patients’ sera predicted poor prognostic outcome [21, 22]. Therefore, the high levels of IGFBP2 in EAC may also have a promising application in diagnosis and / or prognosis in patients with EAC. A comprehensive large size clinical study is needed to clarify this note. In addition, targeting the IGFBP2-EGFR-DNA-PKcs signaling axis may be beneficial in patients with EAC. EGFR inhibitors are used in the treatment of selected cancer patients; especially those with non-small cell lung cancer [52, 53]. A number of experimental DNA-PK inhibitors, such as NU7026, are also under investigation for various cancers [54, 55]. Considering the role of IGFBP2 in forming the complex with EGFR and DNA-PKcs in DNA damage or drug resistance in EAC, the use of EGFR, DNA-PKcs inhibitors or the development of specific IGFBP2 inhibitors will be a promising therapeutic strategy as single agents or in combination with DNA damaging anti-cancer agents.

## Conclusions

We have discovered that IGFBP2 is overexpressed in EAC and ABS induces IGFBP2 probably through degradation of MiRNAs targeting IGFBP2. IGFBP2 protects EAC cells from ABS-induced DNA damage and apoptosis through activating nuclear EGFR-DNA-PKcs pathway. IGFBP2 promotes stabilization of EGFR protein levels and interacts with the EGFR-DNA-PKcs complex to promote DNA repair and cancer cell survival (a graphic summary is shown in Fig. 7c).

## Additional files

Additional file 1: **Table S1.** IGFBP2 siRNAs target sequences. **Table S2.** Information of antibodies used in this study. (PDF 65 kb)

Additional file 2: **Figure S1.** ABS induce IGFBP2, DNA damage and apoptosis in OE19 cells; **Figure S2.** ABS induced IGFBP2 in Barrett’s cells; **Figure S3.** ABS induced MiR-126 degradation in EAC cells; **Figure S4.** HE staining of FLO1 and OE33 in 3D organotypic culture model; **Figure S5.** ABS induce DNA damage and apoptosis in EAC cells; **Figure S6.** Knockdown of IGFBP2 using a second IGFBP2 siRNA; **Figure S7.** Flow cytometry analysis of Annexin V in OE33 cells; **Figure S8.** ABS activate EGFR-DNA-PKcs pathway in EAC cells; **Figure S9.** ABS induce IGFBP2, EGFR and DNA-PKcs nuclear accumulation in OE33 cells; **Figure S10.** IGFBP2 knockdown does not affect EGFR mRNA expression. (PDF 5939 kb)

## Abbreviations

ABS: acidic bile salts
ANOVA: analysis of variance
BE: Barrett’s esophagus
CHX: cycloheximide
co-IP: co-immunoprecipitation assay
DNA-PKcs: DNA-dependent protein kinase, catalytic subunit
DSBs: double-strand breaks
EAC: esophageal adenocarcinoma
EGFR: epidermal growth factor receptor
GERD: gastroesophageal reflux disease
IGFBP2: insulin-like growth factor binding protein 2
IHC: immunohistochemistry
NHEJ: non-homologous end joining
qRT-PCR: quantitative real time reverse transcription-polymerase chain reaction
RIPA: radioimmunoprecipitation
ROS: reactive oxygen species
