# Supplementary material for: Activation of EGFR-DNA-PKcs pathway by IGFBP2 protects esophageal adenocarcinoma cells from acidic bile salts-induced DNA damage
Source: J Exp Clin Cancer Res. 2019 Jan 9;38:13. doi: 10.1186/s13046-018-1021-y (PMC6327430; doi:10.1186/s13046-018-1021-y)
Supplement: Supplementary file 1 — Table S1. IGFBP2 siRNAs target sequences. Table S2. Information of antibodies used in this study. (PDF 65 kb) [file 13046_2018_1021_MOESM1_ESM.pdf]

## Table S1. IGFBP2 siRNAs target sequences

**Santa Cruz Biotechnology (sc-37195, IGFBP2 siRNA)**

sc-37195A:
- Sense: CCGAGUGUCAUCUCUUCUAtt
- Antisense: UAGAAGAGAUGACACUCGGtt

sc-37195B:
- Sense: GAGUGUCAUCUCUUCUACAtt
- Antisense: UGUAGAAGAGAUGACACUCtt

sc-37195C:
- Sense: CCAGUUCUGACACACGUAUtt
- Antisense: AUACGUGUGUCAGAACUGGtt

**Dharmacon (SMARTpool: siGENOME IGFBP2 siRNA)**

siRNA D-010896-06: UGUACAACCUCAAACAGUG
siRNA D-010896-07: GGAGCAGGUUGCAGACAAU
siRNA D-010896-08: GAGCGGAUCUCCACCAUGC
siRNA D-010896-09: CCUCAAGUCGGGUAUGAAG

## Table S2. Antibodies used in this study

| Antibody name | Provider | Cat.# | Application | | | |
|---|---|---|---|---|---|---|
| IGFBP2 | Cell signaling | 3922 | western blotting | IF | IP | IHC |
| EGFR | Invitrogen | MA5-13070 | western blotting | IF | IP | |
| phosphor-EGFR (Tyr1068) | Cell signaling | 3777 | western blotting | | | |
| Caspase 3 | Cell signaling | 9662 | western blotting | | | |
| Cleaved caspase 3 | Cell signaling | 9664 | western blotting | | | |
| PARP | Cell signaling | 9532 | western blotting | | | |
| Cleaved PARP | Cell signaling | 9541 | western blotting | | | |
| H2AX | Cell signaling | 7631 | western blotting | | | |
| phosphor-H2AX (ser139) | Cell signaling | 80312 | western blotting | | | |
| DNA-PKcs | Cell signaling | 12311 | western blotting | | IP | |
| phosphor-DNA-PKcs (Thr2609) | Invitrogen | PA1-29541 | western blotting | IF | | |
| β-Actin | Invitrogen | MA1-744 | western blotting | | | |
