# Supplementary material for: Activation of EGFR-DNA-PKcs pathway by IGFBP2 protects esophageal adenocarcinoma cells from acidic bile salts-induced DNA damage
Source: J Exp Clin Cancer Res. 2019 Jan 9;38:13. doi: 10.1186/s13046-018-1021-y (PMC6327430; doi:10.1186/s13046-018-1021-y)
Supplement: Supplementary file 2 — Figure S1. ABS induce IGFBP2, DNA damage and apoptosis in OE19 cells; Figure S2. ABS induced IGFBP2 in Barrett’s cells; Figure S3. ABS induced MiR-126 degradation in EAC cells; Figure S4. HE staining of FLO1 and OE33 in 3D organotypic culture model; Figure S5. ABS induce DNA damage and apoptosis in EAC cells; Figure S6. Knockdown of IGFBP2 using a second IGFBP2 siRNA; Figure S7. Flow cytometry analysis of Annexin V in OE33 cells; Figure S8. ABS activate EGFR-DNA-PKcs pathway in EAC cells; Figure S9. ABS induce IGFBP2, EGFR and DNA-PKcs nuclear accumulation in OE33 cells; Figure S10. IGFBP2 knockdown does not affect EGFR mRNA expression. (PDF 5939 kb) [file 13046_2018_1021_MOESM2_ESM.pdf]

Fig. S1

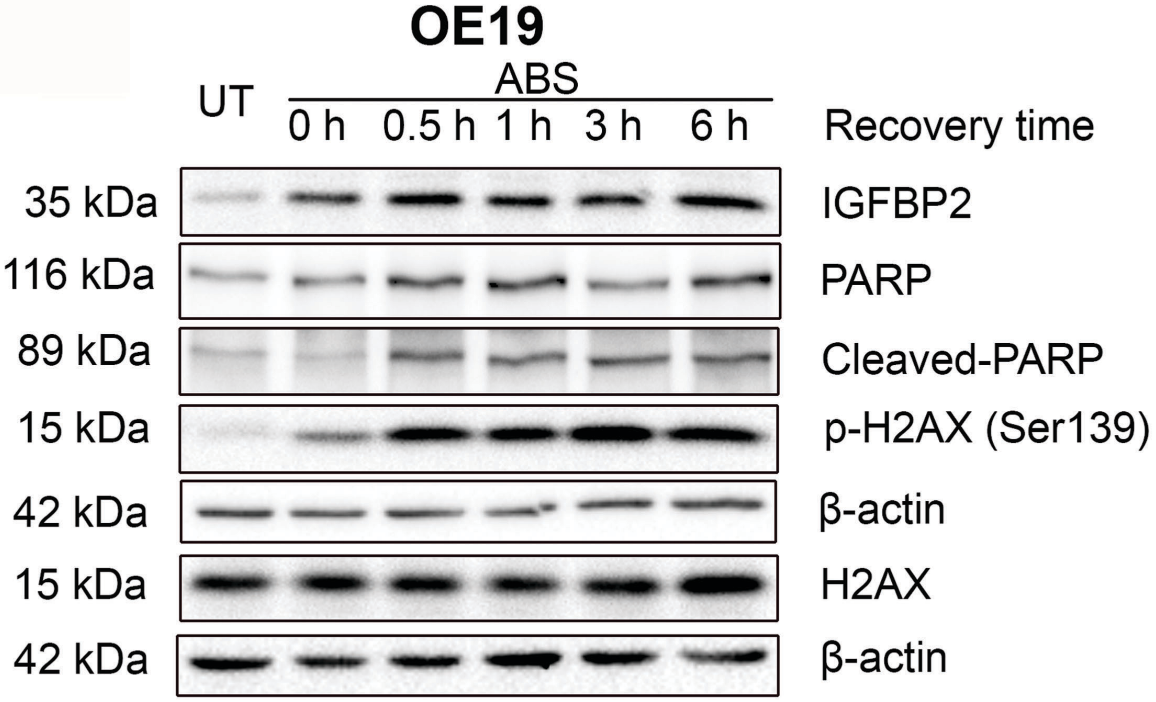

Supplementary Fig. S1. Acidic bile salts induce IGFBP2, DNA damage and apoptosis in OE19 cells. OE19 cells were treated with acidic bile salts (ABS, pH4, 200μM) for 20 minutes and then recovered in the culture medium for desired time points. DNA double strand breaks (p-H2AX) and apoptosis (cleaved PARP) were shown. UT, untreated.

Fig. S2

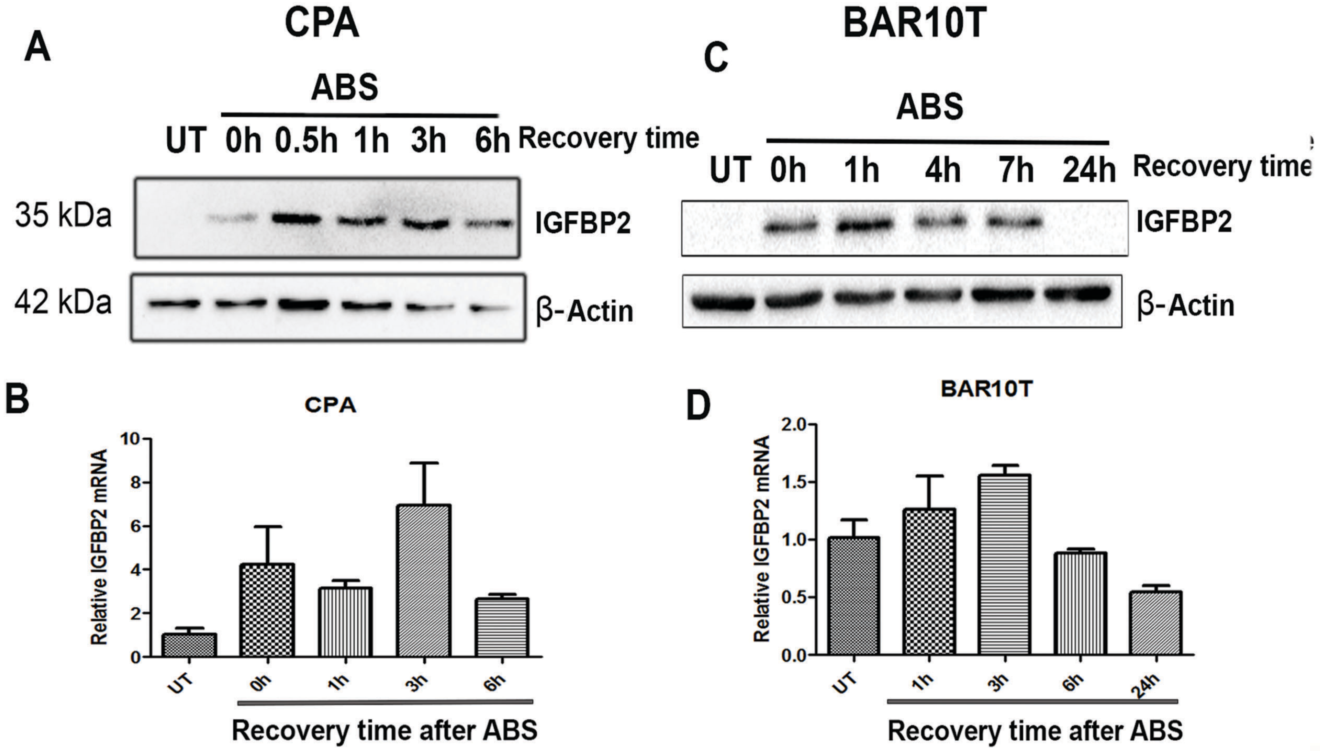

Supplementary Fig. S2. ABS induces IGFBP2 expression in CPA and BAR10T cells. Two Barrett's cell lines, CPA and BAR10T cells were exposed to ABS for 20 min, then recovered for designated time points. Western blotting analyses and qRT-PCR were used to detect protein and mRNA expression levels of IGFBP2 (A and B for CPA, C and D for BAR10T) respectively.

Fig.S3

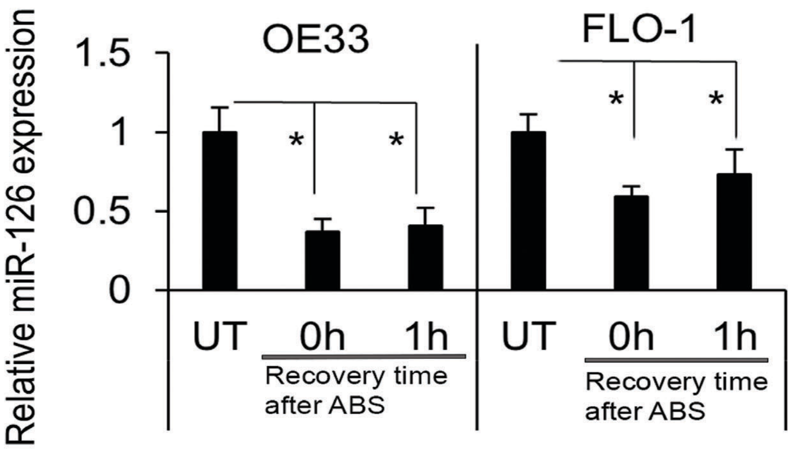

Supplementary Fig. S3. ABS induced MiR-126 degradation in EAC cells. Realtive MiR-126 expression level in OE33 and FLO1 cells after acidic bile salts (ABS) exposure.

Fig. S4

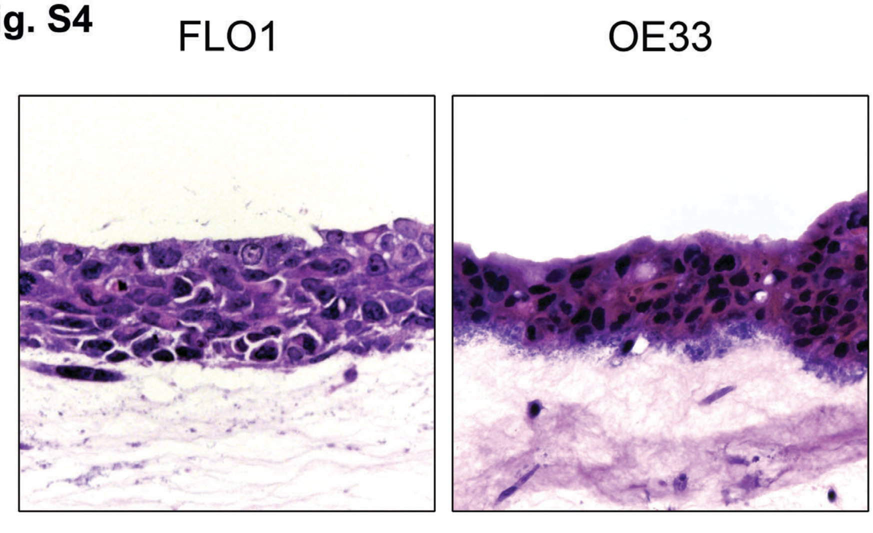

Supplementary Fig. S4. Representative HE staining images of FLO1 and OE33 cells in 3D organotypic culture, showing epithelial cancer cells grew on top of fibroblast cells.

Fig.S5

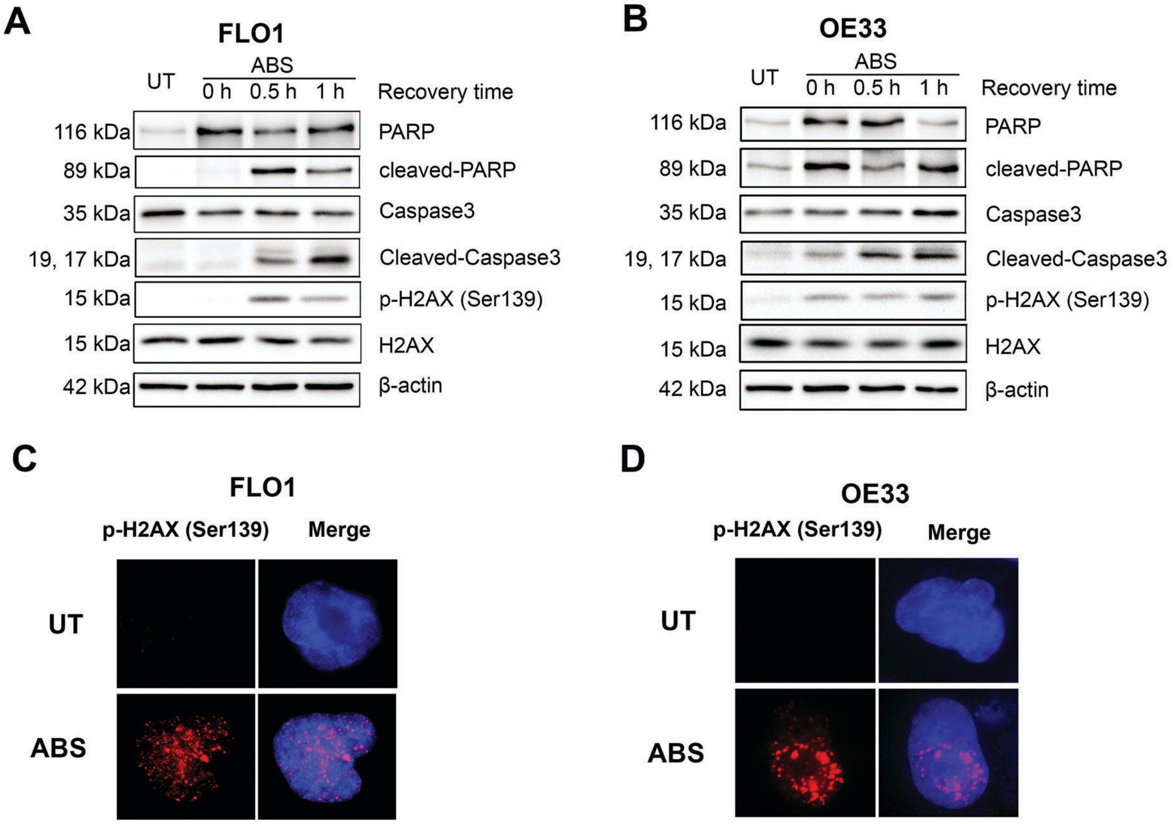

Supplementary Fig. S5. Acidic bile salts induce DNA damage and apoptosis in EAC cells. FLO1 (A) and OE33 (B) cells were treated with acidic bile salts (ABS, pH4, 200μM) for 20 minutes and then recovered in the culture medium for desired time points. Western blot analysis was used to detect DNA double strand breaks (p-H2AX) and apoptosis (cleaved caspase 3 and cleaved PARP). FLO1 (C) and OE33 (D) cells were treated with acidic bile salts (ABS, pH4, 200μM) continuously for 20 minutes and then recovered in the culture medium for 3 hours. Immunofluorescence staining for p-H2AX (Ser139) (red) was performed. DAPI was used to stain nucleus (blue). UT, untreated; ABS, acidic bile salts.

Fig. S6

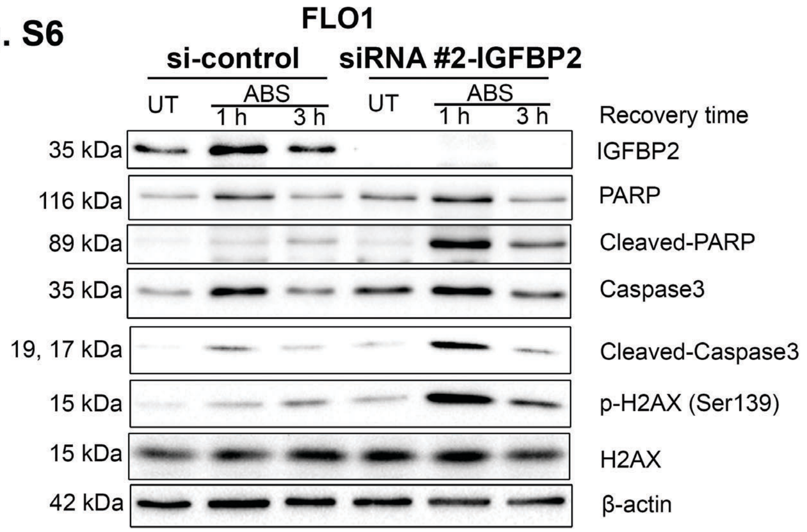

Supplementary Fig. S6 . Knockdown of IGFBP2 using a second IGFBP2 siRNA in FLO1 cells increased acidic bile salts (ABS) -induced DNA damage and apoptosis. To confim IGFBP2 knockdown, we used a second IGFBP2 siRNA from a different company, and treated control and IGFBP2 knockdown cells with ABS. Western blottng analysis were used to detect double strand breaks (p-H2AX) and apoptosis (cleaved caspase3 and cleaved PARP). UT, untreated with ABS.

Fig. S7

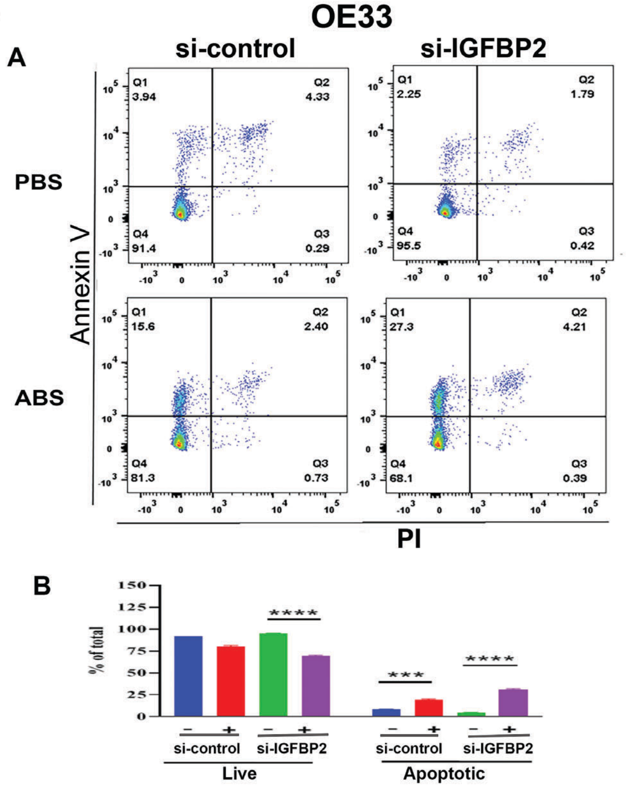

Supplementary Fig.S7. Flow cytometry analysis of Annexin V in OE33 cells. **A,** OE33 cells were transfected with si-IGFBP2 and control siRNA and were exposed to ABS for 30 min, then recovery for 3h. Cells were stained with annexin V and PI and subjected to flow cytometry analysis. **B** shows bar graph of live and apoptotic cells. *** p<0.001, ****p<0.0001.

Fig. S8

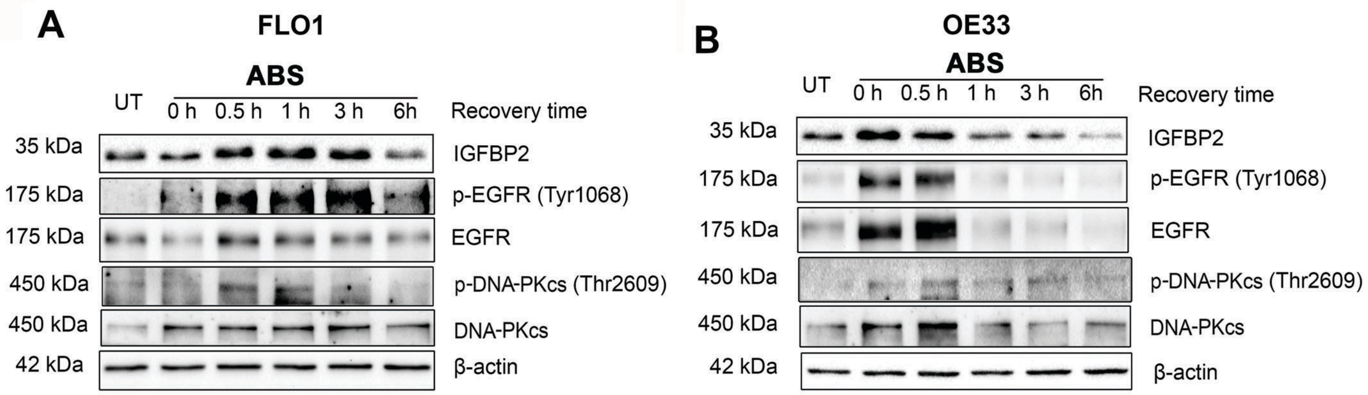

Supplementary Fig. S8. Acidic bile salts activates EGFR-DNA-PKcs pathway in EAC cells. FLO1(A) and OE33 (B) cells were treated with acidic bile salts (ABS, pH4, 200μM) for 20 minutes and then recovered in the culture medium for desired time points. Western blotting assay indicated that p-EGFR (Tyr1068) and p-DNA-PKcs (Thr2609) were induced by ABS treatments in both FLO1 and OE33 cells. Whole cell lysates were applied in western blot. UT, untreated with ABS.

Fig.S9

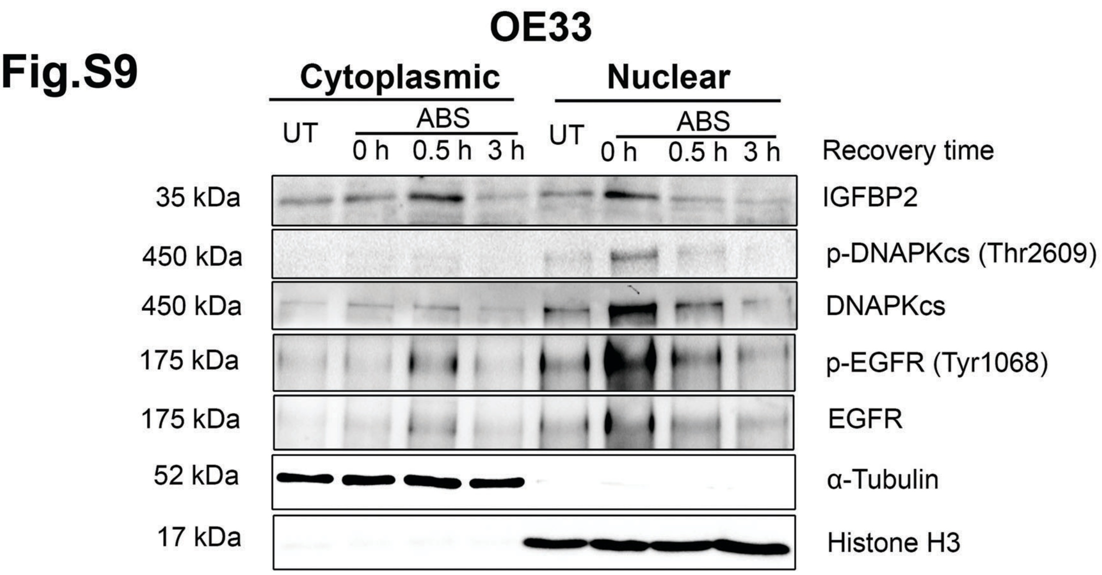

Supplementary Fig. S9. Acidic bile salts induce IGFBP2, EGFR, and DNA-PKcs nuclear accumulation in OE33 cells. OE33 cells were treated with acidic bile salts (ABS, pH4, 200μM) for 20 minutes and then recovered in the culture medium for 0.5 and 3 hours. Cytoplasmic and nuclear fractions were separated and were analyzed using western blotting analysis.

Fig. S10

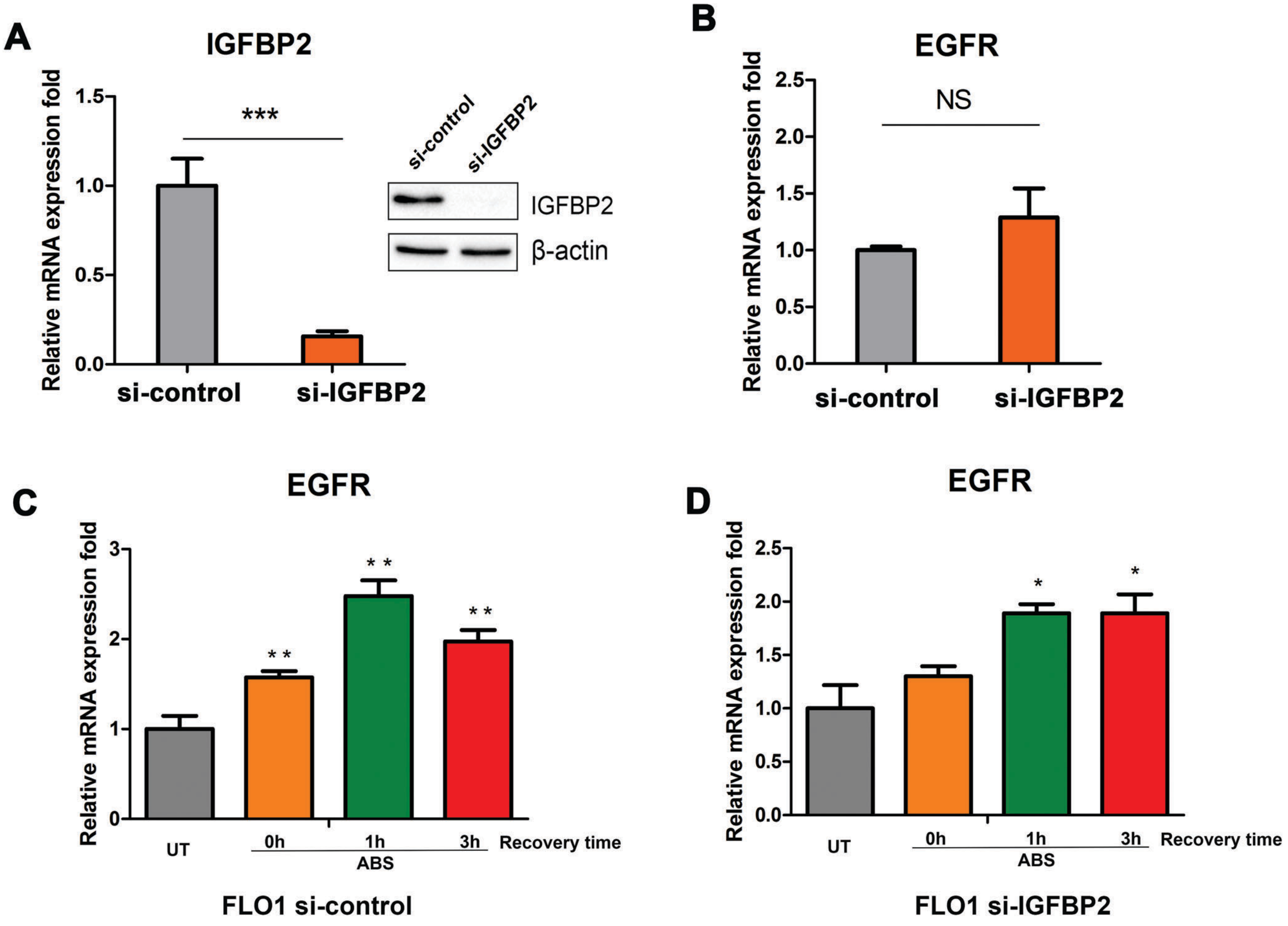

Supplementary Fig.S10. IGFBP2 knockdown does not affect EGFR mRNA expression.
IGFBP2 knockdown in FLO1 cells (A) did not affect EGFR mRNA expression (B). Similarly, IGFBP2 knockdown did not have effect on ABS-induced EGFR mRNA upregulation (C) and (D). NS, not significant; *** $p<0.001$; ** $p<0.01$; * $p<0.05$
